# The crystal structure of D-xylonate dehydratase reveals functional features of enzymes from the Ilv/ED dehydratase family

**DOI:** 10.1038/s41598-018-19192-6

**Published:** 2018-01-16

**Authors:** Mohammad Mubinur Rahman, Martina Andberg, Anu Koivula, Juha Rouvinen, Nina Hakulinen

**Affiliations:** 1 Department of Chemistry, University of Eastern Finland, PO Box 111, FIN-80101 Joensuu, Finland; 2 VTT Technical Research Centre of Finland Ltd, PO Box 1000, FIN-02044 VTT Espoo, Finland

## Abstract

The Ilv/ED dehydratase protein family includes dihydroxy acid-, gluconate-, 6-phosphogluconate- and pentonate dehydratases. The members of this family are involved in various biosynthetic and carbohydrate metabolic pathways. Here, we describe the first crystal structure of D-xylonate dehydratase from *Caulobacter crescentus* (*Cc*XyDHT) at 2.7 Å resolution and compare it with other available enzyme structures from the IlvD/EDD protein family. The quaternary structure of *Cc*XyDHT is a tetramer, and each monomer is composed of two domains in which the N-terminal domain forms a binding site for a [2Fe-2S] cluster and a Mg^2+^ ion. The active site is located at the monomer-monomer interface and contains residues from both the N-terminal recognition helix and the C-terminus of the dimeric counterpart. The active site also contains a conserved Ser490, which probably acts as a base in catalysis. Importantly, the cysteines that participate in the binding and formation of the [2Fe-2S] cluster are not all conserved within the Ilv/ED dehydratase family, which suggests that some members of the IlvD/EDD family may bind different types of [Fe-S] clusters.

## Introduction

There is increasing interest in developing economical and sustainable bioprocesses to convert biomass into valuable organic compounds. Metabolic engineering and synthetic biology have enabled the development of optimized microbial strains for the production of target compounds by taking advantage of known pathways and enzymes. To fully exploit the possibilities offered by nature, a more detailed molecular-level understanding of key metabolic enzymes is required. One important group of these enzymes belongs to the IlvD/EDD protein family, which is involved in the pathways used to produce biofuels such as ethanol, butanol, isobutanol, and 3-methyl-1-butanol as well as platform chemicals such as isobutyric acid, 1,2,4-butanetriol and D-pantothenic acid (vitamin B5)^1–6^.

**Figure 1:**
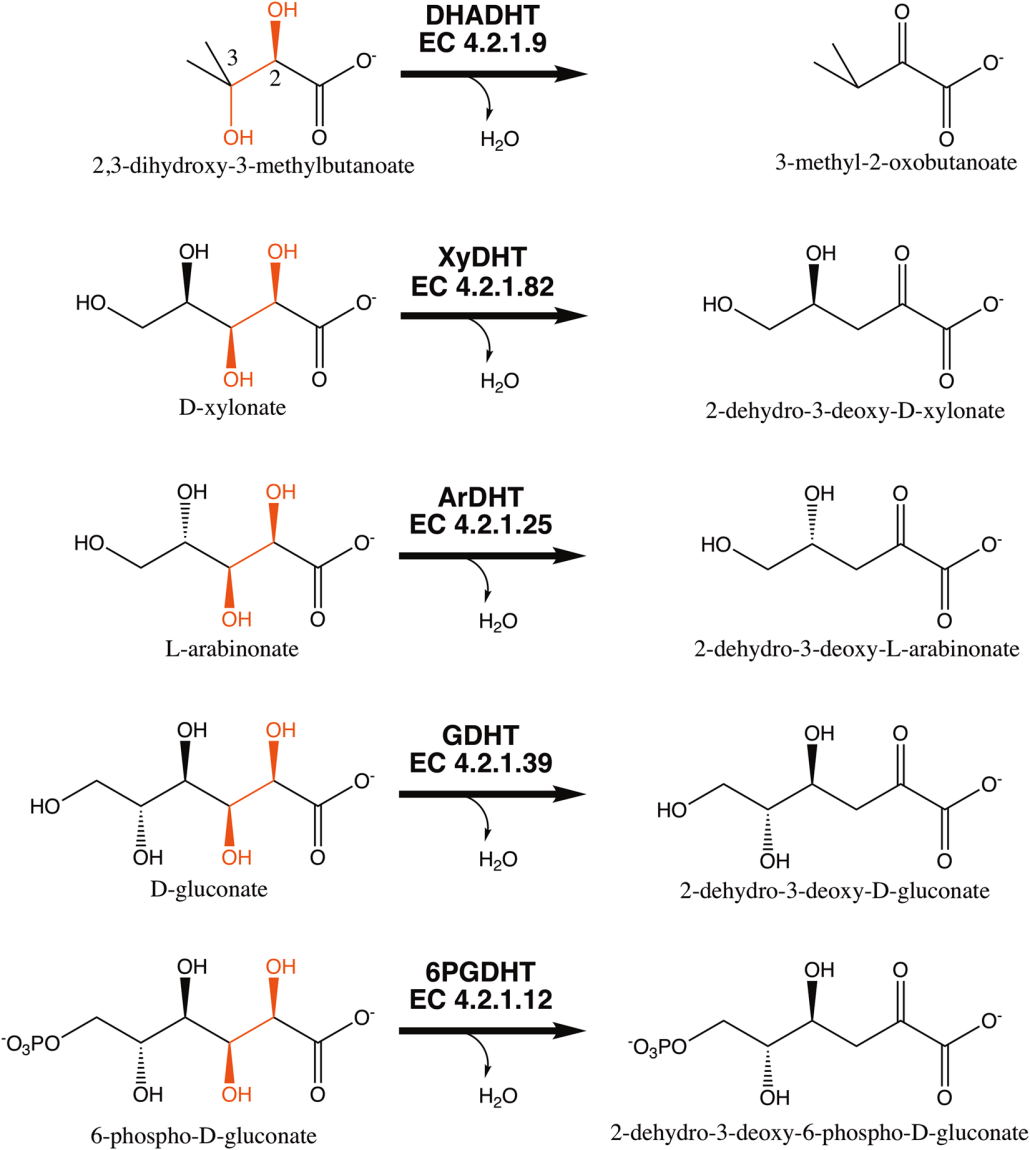
The reactions catalysed by IlvD/EDD enzymes. The identical reactive regions in different substrates are highlighted in red.

Enzymes belonging to the IlvD/EDD protein family are widely distributed among bacteria, archaea, fungi, and algae and in higher plant species^7–12^. The family includes dihydroxy acid dehydratases (IlvD, DHADHT, DHAD; EC 4.2.1.9), which are involved in short-chain amino acid biosynthesis and catalyse the dehydration of 2,3-dihydroxy-3-methylbutanoate^13^, and aldonic acid dehydratases, which are involved in the metabolism of hexose and pentose sugars via the Entner-Doudoroff (ED) pathway or a modified ED pathway^14–18^. Pentonate dehydratases from the IlvD/EDD family participate in the non-phosphorylative oxidation pathways of pentose sugars in which D-xylonate dehydratase (XyDHT) and L-arabinonate dehydratase (ArDHT) catalyse the dehydration of D-xylonate and L-arabinonate, respectively^17–21^. D-gluconate dehydratases (GDHT, GnaD) and 6-phosphogluconate dehydratases (6PGDHT) are involved in the metabolism of glucose via the ED pathway. These two enzymes catalyse the dehydration of gluconate and 6-phosphogluconate, respectively^16,22^. These enzymes all function similarly, by catalysing an elimination reaction in which a hydroxyl group at the C3 position and a proton at the C2 position are cleaved, resulting in the loss of a water molecule (Fig. 1).

Enzymes from the IlvD/EDD family are suggested to contain a [Fe-S] cluster as a co-factor in the active site^19^. Some enzymes from this family have been characterized by using electron paramagnetic resonance (EPR) and UV-Vis spectroscopic methods to explore the structural features of the [Fe-S] cluster and its role in enzyme activity^19,20,23–25^. Based on these studies, two different catalytically active forms of [Fe-S] clusters have been found. A catalytically active [4Fe-4S] cluster that is unstable in aerobic environments has been reported in 6PGDHT from *Zymomonas mobilis*^26^ and in DHADHT from *Escherichia coli*^23^. In contrast, a functional and stable [2Fe-2S] cluster in DHADHTs from several organisms, including bacteria, archaea, fungi and plants, has been observed^24,25^.

A detailed structural and functional characterization of these enzymes is important for understanding the catalytic mechanism and the substrate specificity, which could help improve the synthetic metabolic pathways in biorefinery industries. However, to date, only limited three-dimensional structural information is available for the IlvD/EDD enzymes. The coordinates of *Shewanella oneidensis* 6PGDHT in the apo form have been submitted to the Protein Data Bank (PDB code: 2GP4) but are not yet published. Moreover, this 6PGDHT structure is incompleted and partially incorrect since the original 2GP4 model contains a mixed β-sheet at N-terminal domain, but a corrected model is composed of parallel β-sheet. The model also lacks the cofactor and several surrounding loops in the active site. Recently, we solved the first representative of the IlvD/EDD family in its holo form, an L-arabinonate dehydratase from *Rhizobium leguminosarum* bv *trifolii* (*Rl*ArDHT, PDB code: 5J84). This structure revealed the presence of a [2Fe-2S] cluster and a Mg^2+^ ion in the active site^27^.

In this paper, we describe the first crystal structure of a D-xylonate dehydratase at 2.7 Å resolution and compare it with the two other known crystal structures (PDB codes: 2GP4 and 5J84) belonging to the IlvD/EDD family. D-xylonate dehydratase from *Caulobacter crescentus* (*Cc*XyDHT) shares 42% sequence identity with *Rl*ArDHT, and the crystal structure of *Cc*XyDHT shows that this enzyme has a [2Fe-2S] and a Mg^2+^ ion in its active site, similar to *Rl*ArDHT. The three different enzyme structures available from the IlvD/EDD enzyme family now allow the analysis of the structure and functional features within this protein family.

## Results and Discussion

### The re-refined structure of *So*6PGDHT

The crystal structure of 6-phosphogluconate dehydratase from *Shewanella oneidensis* (*So*6PGDHT) at 2.5 Å resolution (R_work_ = 0.23 and R_free_ = 0.29) is available in the Protein Data Bank (PDB code: 2GP4), but the structure lacks a [Fe-S] cluster and a divalent metal ion. When inspecting the electron density map of *So*6PGDHT, we found several obscured regions in the crystal structure. Therefore, the 2GP4 model was re-refined, which resulted in improved R-values (R_work_ = 0.17 and R_free_ = 0.23). However, the improved model of 6PGDHT still had missing regions, Val35-Leu63, Lys182-Ile185 and Thr215-Thr226, and it was lacking density for the [Fe-S] cluster or Mg^2+^ ion in the active site. The re-refinement resulted in new locations for the active site residues Cys112 and Asp113. In the re-refined model, the putative iron-sulphur cluster binding residues Cys112 and Cys154 are sufficiently close to ligate a [Fe-S] cluster. The location of the third putative cluster-binding residue Cys220 is undefined because, it is located in the missing loop, the structure of which is unknown due to the absence of electron density.

### Overall structure of *Cc*XyDHT

The crystal structure of holo-*Cc*XyDHT was determined at 2.7 Å resolution by molecular replacement, using L-arabinonate dehydratase from *Rhizobium leguminosarum* bv *trifolii* (5J84) as a template. The final structure had good R-factor and R-free values of 17.8% and 22.3%, respectively. The structure contained a tetramer in the asymmetric unit in the space group *C*2. Unambiguous electron density was seen for all of the amino acid residues of *Cc*XyDHT, except for the disordered region at the N-terminus, which includes Asp1-Ser2 and the N-terminal Strep-tag II. The refinement resulted in a final model with good refinement statistics (Table 1).

**Table 1:** Data collection and refinement statistics. The structure was determined from a single crystal. The value in parentheses are for the highest-resolution shell.

Data collection	*Cc*XyDHT
Beam line	I04, DLS, England
Wavelength (Å)	0.979497
Resolution (Å)	59.12 − 2.66 (2.76 − 2.66)
Space group	*C* 1 2 1
Cell dimension	
a, b, c (Å)	270.41, 236.14, 65.17
α, β, γ (°)	90, 97.38, 90
Total reflections	393801 (60419)
Unique reflections	115354 (11560)
Multiplicity	3.4 (3.4)
Completeness (%)	99.54 (99.80)
Mean I/σ (I)	17.2 (2.1)
Wilson B-factor (Å^2^)	59.91
R-meas (%)	7.0 (82.1)
*CC*_1/2_	99.9 (95.8)
**Refinement statistics**	
Reflections used in refinement	110218 (10961)
Reflections used for R-free	5509 (546)
R-work	0.1769 (0.3295)
R-free	0.2174 (0.3814)
Number of non-hydrogen atoms	18336
Protein	17992
Solvent	324
Fe/S (cluster)	8/8
Mg	4
Protein residues	2354
**RMS deviations**	
Bond length (Å)	0.007
Bond angles (°)	0.95
**Ramachandran analysis (%)**	
Favoured	95.6
Allowed	3.9
Outliers	0.5
Rotamer outliers	0.0
Clashscore	8.33
**Average B-factor (Å** ^**2**^ **)**	67
Protein	68
Solvent	58
Fe/S (cluster)	79/71
Mg	83
**PDB ID**	**5OYN**

**Figure 2:**
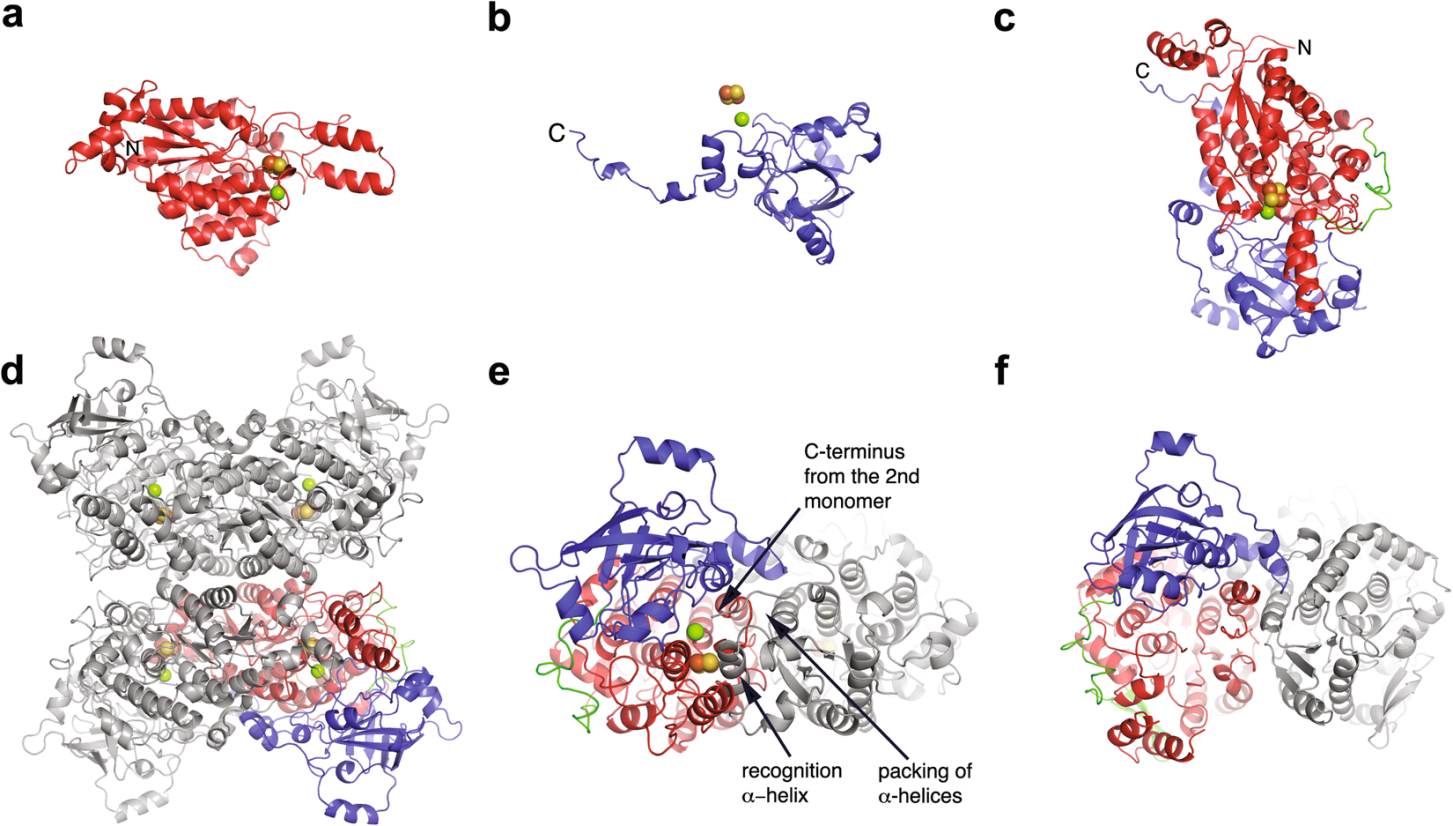
Structure of *Cc*XyDHT and comparison of the dimers; the N- and C-terminal domains are shown in red and in blue, respectively. A long loop connecting the domains is shown in green. The [2Fe-2S] cluster is shown with yellow and orange spheres, and Mg^2+^ is shown as a green sphere. (**a**) N-terminal domain, (**b**) C-terminal domain, (**c**) monomer, (**d**) tetramer, (**e**) the dimer of *Cc*XyDHT in which the monomeric unit is shown in colours and the dimeric counterpart is in grey, and (**f**) the dimer of *So*6PGHDT. The key features on the monomer-monomer interface are marked with arrows.

The enzyme is an α/β protein that consists of two domains: a N-terminal domain (residues Asn3-Leu358) and a C-terminal domain (residues Leu383-His591) (Fig. 2a,b). The two domains are connected by a long loop from Gln359 to Phe382. The N-terminal domain is composed of a β-sheet with four parallel β-strands surrounded by four α-helices. In addition, the N-terminal domain contains an extension at the N-terminus (residues Arg10 - Ser43) with three α-helices and an insertion with β-hairpin (residues Gly157 – Val164) and a helix-loop-helix (residues Thr168 – Ser194). The C-terminal domain is composed of a β-sheet consisting of six parallel and two anti-parallel β-strands that are arranged like a β-barrel. Secondary structure analysis showed that the complete polypeptide chain consists of 27 α-helices and 16 β-strands. Each monomer contains one [2Fe-2S] cluster and a Mg^2+^ ion. The substrate binding site is located in the cavity between the domains, but the [Fe-S] cluster and the Mg^2+^ binding site are located entirely in the N-terminal domain (Fig. 2c).

### Quaternary structure

The quaternary structure of *Cc*XyDHT is a homotetramer (Fig. 2d), similar to the homotetrameric structure of *Rl*ArDHT. D-gluconate dehydratase from *Achromobacter xylosoxidans* has also been reported to form a tetramer in solution^22^. A packing analysis of the crystal structure using the PISA server^28^ suggested that the quaternary structure contains a stable dimer with an extensive monomer-monomer interface (4640 Å^2^). This interface is predominantly formed by residues from the larger-sized N-terminal domain. An α-helix (residues 99–115) from this domain forms the central part of the interface. The tight dimer then packs against another similar dimer in such a way that one monomer has an interface with both polypeptide chains from the second dimer (887 and 607 Å^2^). The total monomer-monomer interface area in the tetramer is 12268 Å^2^.

In contrast, *So*PGDHT forms a dimer in the crystal structure that is similar to the tight dimer of *Cc*XyDHT (Fig. 2e,f). However, unlike *Cc*XyDHT, *So*PGDHT does not form a tetrameric structure. Many IlvD/EDD enzymes such as L-arabonate dehydratase from *Azospirillum brasiliense*^20^, dihydroxy acid dehydratase from *Spinacia oleracea*^24^, and 6-phosphogluconate dehydratase from *Zymomonas mobilis* have been reported to form dimers in solution^29^.

The formation of the extensive dimer interface in *Cc*XyDHT (and in *Rl*ArDHT) is essential for the formation of the active site. Although the residues that participate in the binding of Mg^2+^ and the [2Fe-2S] cofactor are located in the N-terminal domain of the first monomer, the polypeptide chain from the second monomer covers the active site by extending its N-terminal helix (residues 21–33) into the first monomer. Because the amino acid residues of this helix, which point towards the active site, are different in *Cc*XyDHT and *Rl*ArDHT, this helix may be called a substrate recognition helix. In addition, the C-terminus of the dimeric counterpart protrudes into the active site of the first monomer by placing His591, which forms a salt bridge with Glu463, in the active site (Fig. 2e). The crystal structure of *So*6PGDHT shows similar dimer formation to that observed in *Cc*XyDHT but does not show any evidence that the polypeptide chain from the dimeric counterpart participates in the formation of the active site. In contrast, the *So*6PGDHT structure represents the apo form, and some of the loop regions are not visible in the electron density map due to disorder in the absence of cofactor (Fig. 2f).

### Structural comparison of three IlvD/EDD enzymes

**Figure 3:**
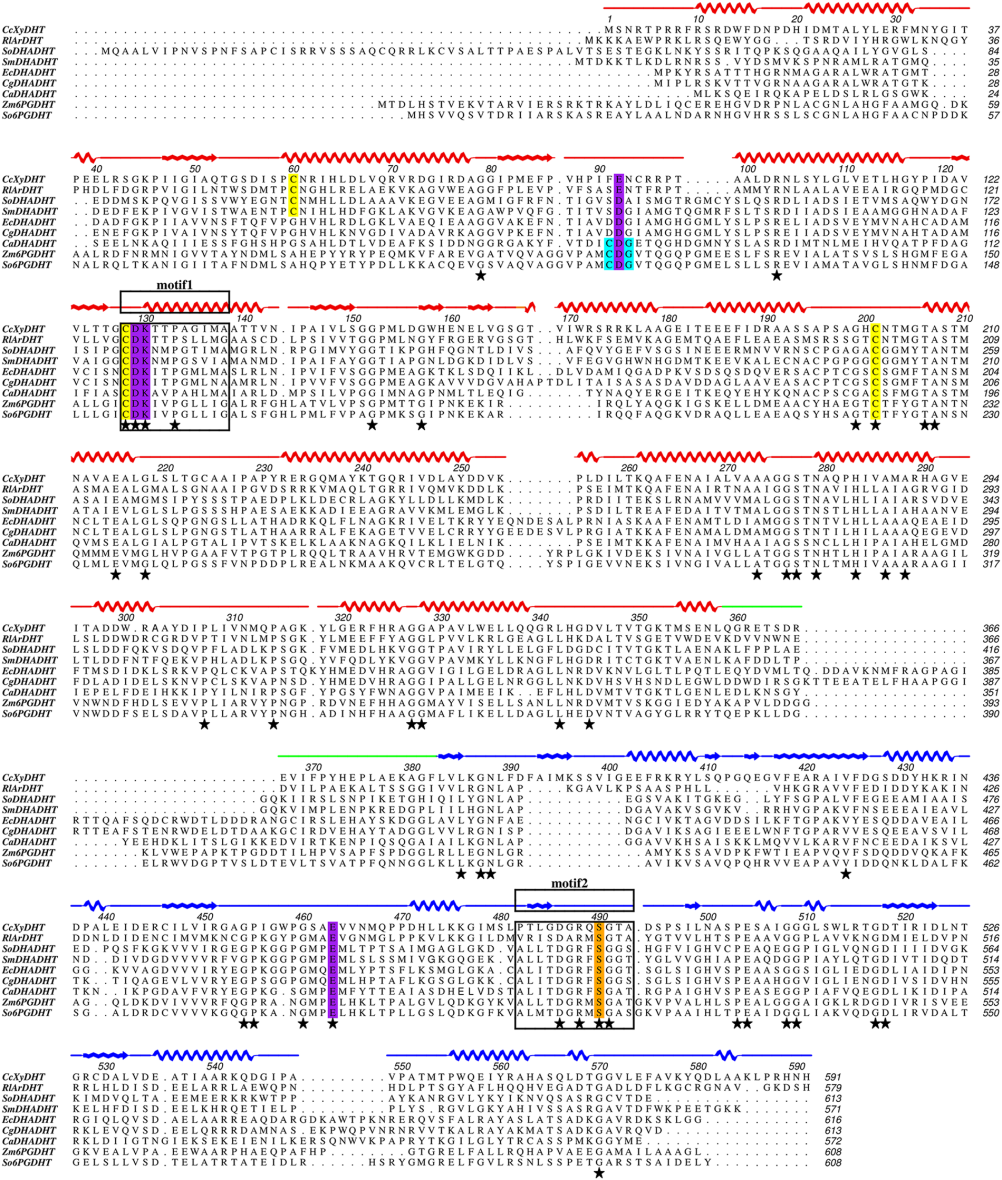
Multiple sequence alignment of IlvD/EDD enzymes. *Cc*XyDHT is a D-xylonate dehydratase from *Caulobacter crescentus* (GeneBank: ANS60449), *Rl*ArDHT is an L-arabinonate dehydratase from *Rhizobium leguminosarum* bv. *trifolii* (GeneBank: ANS60454), *Sm*DHADHT is a dihydroxy acid dehydratase from *Streptococcus mutans* (GeneBank: KZM62800), *So*DHADHT is a dihydroxy acid dehydratase from *Spinacia oleracea* (GeneBank: KNA20834), *Ec*DHADHT is a dihydroxy acid dehydratase from *Escherichia coli* (GeneBank: CDZ22543), *Cg*DHADHT is a dihydroxy acid dehydratase from *Corynebacterium glutamicum* (GeneBank: BAV23085), *Ca*DHADHT is a dihydroxy acid dehydratase from *Clostridium acetobutylicum* (GeneBank: WP_010966867), and *Zm*6PGDHT and *So*6PGDHT are 6-phosphogluconate dehydratases from *Zymomonas mobilis* (GeneBank: WP_013934127) and *Shewanella oneidensis* (GeneBank: WP_011072452). The N- and C-terminal domain are shown in red and blue, connecting loop in green. The [2Fe-2S] cluster coordinating residues are shown in yellow, Mg^2+^ ion coordinating residues are in magenta and serine that play role as Lewis base is shown in orange, respectively. Cys-Asp-Gly motif is shown in cyan. Black stars represent the fully conserved residues.

**Figure 4:**
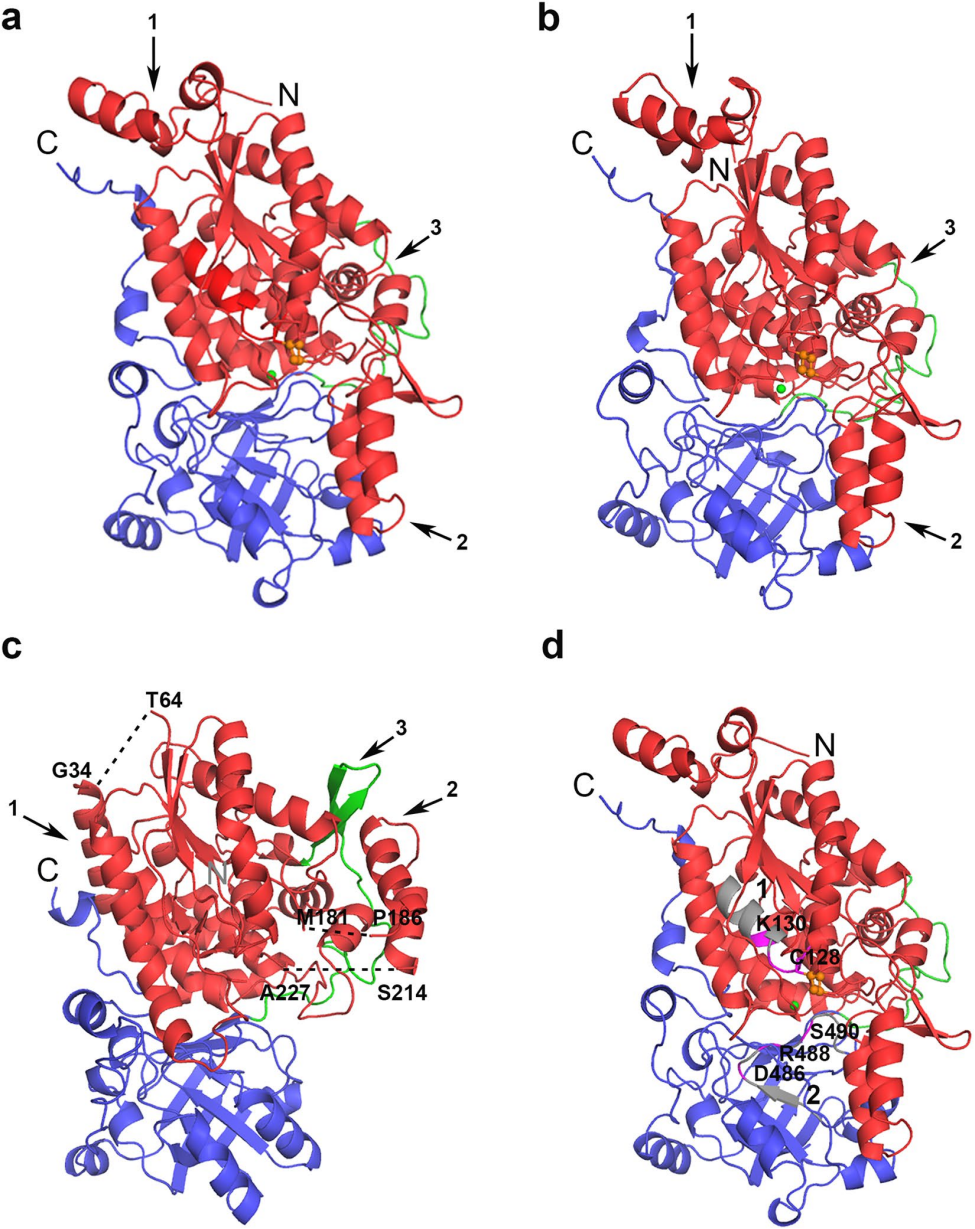
The monomeric structures of IlvD/EDD enzymes: (**a**) *Cc*XyDHT, (**b**) *Rl*ArDHT, and (**c**) *So*6PGDHT. The N-terminal domain is in red, the C-terminal domain in blue, and the connecting region is shown in green. The undefined regions in the structure of *So*6PGDHT are shown as dashed lines. The arrows indicate the major differences between the structures: 1) the N-terminal region, 2) the flipped helix-loop-helix structure, and 3) the β-hairpin insertion of *So*6PGDHT in the connecting region. (**d**) The location of sequence motifs 1 and 2 in the *Cc*XyDHT monomer. The conserved residues in both motifs are in purple, and other residues are in grey.

The sequence of *Cc*XyDHT is 42% identical to *Rl*ArDHT and 27% identical to *So*6PGDHT. A sequence alignment of these three enzymes and of representative members of the IlvD/EDD family is shown in Fig. 3. The higher sequence identity between *Cc*XyDHT and *Rl*ArDHT, compared to *So*6PGDHT, is also reflected in a low, 0.70 Å RMS deviation between the Cα atoms of monomer proteins. The overall structures are very similar except for a small insertion of a β-hairpin at Leu409-Val417 in *Cc*XyDHT. The [2Fe-2S] cluster and Mg^2+^ ion coordinating residues are well conserved between these enzymes. The improved model of *So*6PGDHT can be superimposed with *Cc*XyDHT with a RMSD of 1.95 Å. The N-terminal core domain consisting of a four-stranded parallel β-sheet surrounded by α-helices and the C-terminal core containing a mixed eight-stranded β-sheet are similar. However, the large RMSD value between *So*6PGDHT and *Cc*XyDHT is due to the following major differences between the structures: 1) the N-terminal regions, 2) the helix-loop-helix motifs and 3) the β-hairpin structures (Fig. 4).

The N-termini of *Cc*XyDHT and *So*6PGDHT are very different. In *Cc*XyDHT, this region (residues 1–45) forms a bundle of three short helices, but in *So*6PGDHT, this region is longer (residues 1–65) and contains two long α-helices, which have a very different orientation and pack against the core protein in an extended fashion. A part of the N-terminal region (35–63) is undefined in the structure of *So*6PGDHT. Perhaps the most remarkable structural difference between the proteins is the location of the Pro186-Ser214 region that contains a helix-loop-helix motif structure at the N-terminal domain of *So*6PGDHT. The helix-loop-helix in *So*PGDHT has been flipped and moves by approximately 30 Å from the position observed in *Cc*XyDHT (and in *Rl*ArDHT), where it partially covers the active site. Interestingly, in an open form of an *Rl*ArDHT mutant co-crystallized with L-arabinonate, this helix-loop-helix structure is slightly displaced by 7 Å, which suggests that it has a functional role in the catalytic cycle^27^.

In *So*6PGDHT, the helix-loop-helix region is twisted away from the active site. Unfortunately, the preceding loop (residues 182–185) and the following loop (residues 215–226) regions are disordered in the *So*6PGDHT structure, so how accessible the active site of *So*6PGDHT is compared to those of *Cc*XyDHT and *Rl*ArDHT remains an open question. The third difference between *So*6PGDHT and *Cc*XyDHT is an additional β-hairpin structure (residues 383–396) in the connecting loop of *So*6PGDHT. This packs against the abovementioned helix-loop-helix structure (Fig. 4a–c).

### Sequence motifs to identify the IlvD/EDD family

We have analysed the multiple amino acid sequence alignment of some representative members of the IlvD/EDD family. The sequence alignment is presented in Fig. 3, including the sequences of the three enzymes (*Cc*XyDHT, *Rl*ArDHT, *So*6PGDHT) for which three-dimensional structure information is available. The description of the IlvD/EDD protein family in the PROSITE database^30^ reports that this family has two common sequence motifs. The first sequence motif consists of eleven residues with a peptide pattern Cys-Asp-Lys-x(2)-Pro-[Gly/Ala]-x(3)-[Gly/Ala], where Cys-Asp-Lys and Pro are conserved residues. The second sequence motif consists of twelve residues with a peptide pattern [Ser/Gly/Ala/Leu/Cys]-[Leu/Ile/Met/Phe]-[Leu/Ile/Val/Met/Phe]-Thr-Asp-[Gly/Ala]-Arg-[Leu/Ile/Val/Met/Phe/Tyr]-Ser-[Gly/Ala]-[Gly/Ala/Val]-[Ser/Thr], where Thr-Asp, Arg and Ser (without brackets) are conserved residues.

These two motifs can be found in the *Cc*XyDHT structure (Figs 3, 4d). The first motif is Cys-Asp-Lys-Thr-Thr-Pro-Ala-Gly-Ile-Met-Ala at the N-terminal domain (residues 128–138). These eleven residues are located in the helix α6 and the loop connecting the strand β3 and helix α6. The conserved Cys128 participates in the binding of the [2Fe-2S] cluster, Asp129 and Lys130 (in carbamylated form) in the binding of Mg^2+^.

The second motif is Pro-Thr-Leu-Gly-Asp-Gly-Arg-Gln-Ser-Gly-Thr-Ala (residues 482–493) in *Cc*XyDHT. These twelve residues are located at the C-terminal domain consisting of strand β13 and the loop follows. Interestingly, the first two and last two residues of this motif in *Cc*XyDHT do not match the predicted sequence motif. The proposed conserved threonine is not conserved in *Cc*XyDHT and is replaced by Gly485. The conserved Asp486 forms hydrogen bonds with the main chain nitrogens of Gly452 and Ala453 in the loop structure. The conserved Arg488 is located rather close (4.3 Å) to the Mg^2+^ but probably does not directly participate in the binding of magnesium; consequently, its role may be electrostatic. Ser490, which is proposed to act as a base in enzyme catalysis, as supported by experimental results from the corresponding Ser480Ala mutant of *Rl*ArDHT, is fully conserved among the IlvD/EDD protein family^27^.

### The active site of *Cc*XyDHT

The subsequent analysis of the active site is based solely on the crystal structures of the holo forms of *Cc*XyDHT and *Rl*ArDHT and not on the structure of *So*6PGDHT because the latter structure represents an incomplete structure in the apo form. The electron density map of the *Cc*XyDHT crystal structure shows that the binding of the Mg^2+^ and the [2Fe-2S] cluster to the active site is similar to that observed in *Rl*ArDHT^27^.

**Figure 5:**
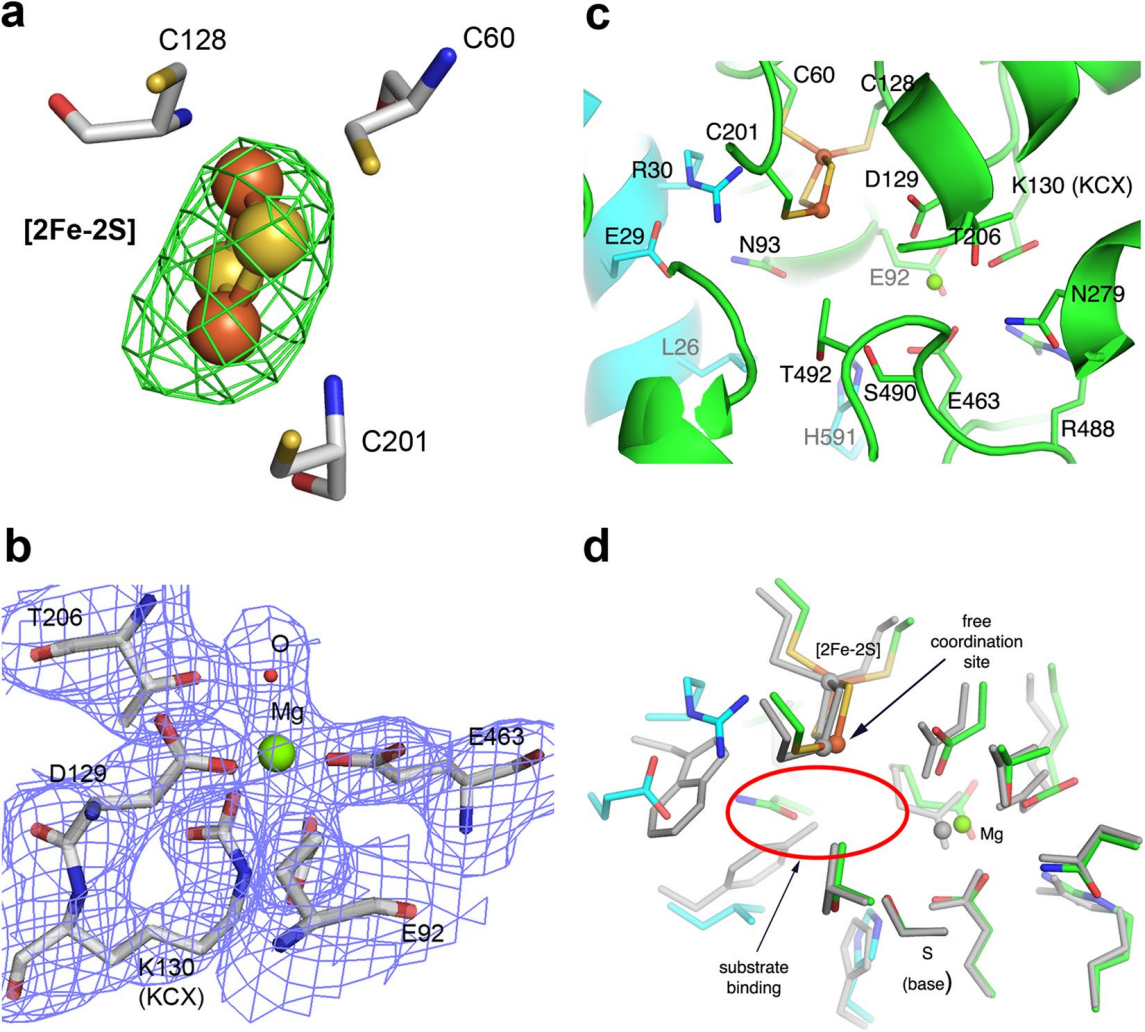
The active site of *Cc*XyDHT. (**a**) The mFo-DFc omit maps (green) contoured at 3σ level for the [2Fe-2S] cluster; and (**b**) The 2mFo-DFc electron density maps (blue) contoured at 1σ level for the Mg^2+^ ion and the binding residues; (**c**) the active site residues, which participate in the binding of the cofactors and the putative substrate binding residues are shown. The protein backbone cartoon colour is green for the first monomer and cyan for the second monomer; (**d**) The superimposition of *Cc*XyDHT (in green and cyan) and *Rl*ArDHT (grey). The most likely binding site for the substrate is circled with a red ellipse.

The hexacoordination of the Mg^2+^ ion resembles a square bipyramid in which Glu92, Asp129, Glu463 and a water (or hydroxide ion) are approximately in the plane and another water molecule (or a hydroxide ion) and carbamylated Lys130 are in the axial positions. One water molecule in the square plane is hydrogen-bonded (2.7 Å) to Thr206. Due to the obtained resolution, we cannot fully exclude the possibility that Thr206 may also directly interact with the Mg^2+^ ion. However, the observed distance from Thr206 to the Mg is approximately 3.8 Å. Identical residues can be found in the structure of *Rl*ArDHT and all four Mg^2+^ binding residues (and Thr206) are fully conserved within the protein family (Fig. 3), suggesting that the binding of magnesium is a universal feature of the IlvD/EDD enzyme family. The crystal structure of *Cc*XyDHT unambiguously shows that Lys130 is carbamylated (Fig. 5c,d) as is also found in the crystal structure of *Rl*ArDHT^27^. The residues Asp129 and Lys130 (or more correctly KCX130) belong to the N-terminal sequence motif. The complete structures of *Cc*XyDHT and *Rl*ArDHT support an interpretation that carbamylation of this lysine is a common feature among IlvD/EDD enzymes.

The crystal structure of *Cc*XyDHT suggests that the enzyme has a [2Fe-2S] cluster, similar to that observed in *Rl*ArDHT. In the cluster, the Fe1 atom is coordinated by two sulfide ions and two cysteines (Cys60 and Cys128). Cys128 belongs to the Cys-Asp-Lys motif, which is fully conserved among IlvD/EDD enzymes. Interestingly, Cys60 is conserved only among the subfamily of IlvD/EDD enzymes including pentonate dehydratases and some dihydroxy acid dehydratases^19^. In 6PGDHT, the putative corresponding cysteine belongs to a Cys-Asp-Gly motif (Fig. 1), which is not observed in pentonate dehydratases. This may reflect the organization of a different cluster type, for example, the binding of [2Fe-2S] versus [4Fe-4S] in the active site. In *Cc*XyDHT, Fe2 atom of the cluster is coordinated by two sulfide ions and one cysteine residue (Cys200). This cysteine residue is fully conserved within the IlvD/EDD protein family.

**Figure 6:**
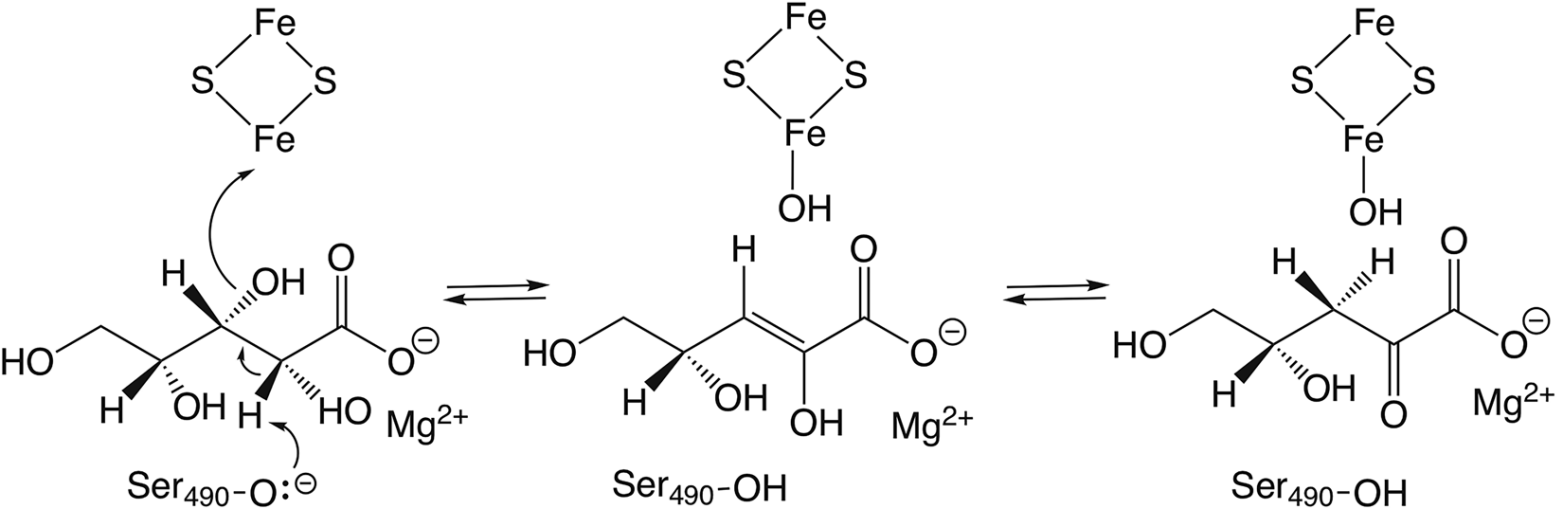
Proposed reaction mechanism for D-xylonate dehydratase.

Residues Ser490 and Thr492 from the C-terminal sequence motif participate in the formation of the substrate binding site (Fig. 5c,d). Based on the crystal structure and mutagenesis studies of *Rl*ArDHT, we have previously suggested that serine acts as a Lewis base in catalysis^27^. In *Cc*XyDHT, Ser490 is approximately 6 Å away from the [2Fe-2S] cluster and 4 Å away from the Mg^2+^ ion. As in *Rl*ArDHT, OG1 of Thr492 forms a short (and strong) hydrogen bond (2.6 Å) with OG of Ser490 in *Cc*XyDHT. In addition, a hydrogen bond (3.2 Å) exists between the main chain nitrogen of Thr492 and OG1 of Ser490. Furthermore, NE1 of Trp171 is hydrogen bonded (2.9 Å) to OG1 of Thr492. This hydrogen bond network may increase the nucleophilicity of Ser490. Thr492 in *Cc*XyDHT and the corresponding Thr482 in *Rl*ArDHT are not conserved within the IlvD/EDD protein family (replaced by Ala/Gly) as seen in Fig. 3. However, the next amino acid after Ala/Gly in the sequence within the members of this protein family is Ser/Thr, which could play a similar role to Thr492 in the *Cc*XyDHT structure. The proposed elimination reaction is shown in Fig. 6, where [2Fe-2S] cluster act as a Lewis acid and the alkoxide ion form of Ser490 is a base. In the end, the enol product is tautomerized to the final C2 oxo product.

### Molecular determinants for the substrate specificity

To understand the substrate specificity between *Cc*XyDHT and *Rl*ArDHT, the active sites were superimposed. The preferred substrates for *Cc*XyDHT and *Rl*ArDHT are stereoisomers of pentonates, having different configurations at the C4 position. The K_m_ values for D-xylonate and L-arabinonate are in the mM range (2–10 mM) for both enzymes, but the k_cat_ values differ more significantly. The catalytic efficiency k_cat_/K_m_ is 120 times higher in *Cc*XyDHT for D-xylonate compared to L-arabinonate, and 10 times higher in *Rl*ArDHT for L-arabinonate compared to D-xylonate. In addition to pentonate sugar acids, various hexonate sugar acids can be dehydrated, as long as the configurations at C2 and C3 are maintained^19^.

The active site superimposition of *Cc*XyDHT and *Rl*ArDHT shows that the binding of the Mg^2+^ and the [2Fe-2S] cluster is very similar, even identical in both enzymes. However, the differences in the active site cleft are significant in the N-terminal helix (residues 21–33) from the dimeric counterpart. The N-terminal helix can be therefore identified as a substrate recognition helix, where Trp31 in *Rl*ArDHT is replaced by Arg30 in *Cc*XyDHT. This Arg residue forms a salt-bridge with Glu29 in *Cc*XyDHT (the corresponding residue in *Rl*ArDHT is Gly30). Tyr27 in *Rl*ArDHT is replaced by Leu26 in *Cc*XyDHT, and consequently, the conformation of Asn92 is altered. In addition, both enzymes have a C-terminal histidine residue from the dimeric counterpart (His591 in *Cc*XyDHT and His579 in *Rl*ArDHT) in the active site (Fig. 5d). The carboxylate group of the C-terminus is salt-bridged to Arg22 and Lys422 in *Rl*ArDHT and to Arg174 in *Cc*XyDHT. The differences between the catalytic efficiency thus probably originate from the recognition helix and C-terminus, which highlights the important role of the dimerization.

## Materials and Methods

### Protein purification and crystallization

D-xylonate dehydratase from *Caulobacter crescentus* was heterologously expressed in *E. coli*, purified, and crystallized, and data were collected at 2.66 Å resolution using synchrotron radiation as described previously^19,31^. The data were processed and scaled using XDS and XSCALE^32^.

### Structure determination and refinement

A crystal structure of *Cc*XyDHT at 2.7 Å was determined by molecular replacement using PHASER^33^ with the coordinates of L-arabinonate dehydratase (PDB code 5J84) as a template. The model building was performed in COOT^34^. The refinement of the model was performed using PHENIX refine^35^. The validation of the refinement was done using MolProbity^36^. The re-refinement of *So*6PGDHT (PDB code 2GP4) was also done using the PHENIX software^35^.

### UV-Vis spectroscopy

The presence of a [Fe-S] cluster in *Cc*XyDHT was analysed using UV-Vis spectroscopy. A purified protein sample was diluted with a sample storage buffer consisting of 50 mM Tris-HCl and 5 mM MgCl_2_ at pH 7.5. The sample was loaded into a quartz cuvette and data were collected using a UV-Vis/NIR/900 spectrophotometer (PerkinElmer, USA) at wavelengths of 260–800 nm against sample storage buffer. The spectrum is shown in Figure S1.

### Enzyme activity test

The *Cc*XyDHT activity was assayed as described previously^19^. The purified enzyme (6 µg in a 600 µl reaction) was incubated at 30 °C in 50 mM Tris-Cl, 10 mM MgCl_2_, at pH 8.5 using 20 mM D-xylonate as substrate. At 2-min intervals, a 100-μl sample was transferred to a microcentrifuge tube and the reaction was stopped by adding 10 μl of 12% trichloroacetic acid (TCA). The product formation was measured using the thiobarbituric acid (TBA) assay^37^. Aliquots of 50 μl from all samples were transferred into fresh microcentrifuge tubes, after which 125 μl of 25 mM periodic acid (dissolved in 0.2 M H_2_SO_4_) was added and incubated at room temperature for 20 min. To the reaction, 250 μl of 2% sodium arsenate in 0.5 M HCl was added and mixed, followed by 1 ml of 0.3% TBA. The samples were then incubated at 100 °C for 10 min. Prior to reading the absorbance at 549 nm, the samples were mixed with an equal volume of DMSO.

### Sequence alignment and structural comparisons

The amino acid sequences of the enzymes were collected from the NCBI GeneBank^38^. The sequence alignment was carried out using the online multiple sequence alignment tools Clustal Omega^39^ and MacVector^40^. The secondary structure analysis was carried out using the online tool STRIDE^41^. The alignment figure was created using ALINE^42^. The superimposition of the crystal structures and the figures of the three-dimensional enzyme structures were made using PyMOL^43^.

### Data availability

The atomic coordinates and structure factors have been deposited in the Protein Data Bank under the accession code 5OYN.

## Electronic supplementary material

Supplementary Information

## References

[1] Lang, K., Zierow, J., Buehler, K. & Schmid, A. Metabolic engineering of Pseudomonas sp. strain VLB120 as platform biocatalyst for the production of isobutyric acid and other secondary metabolites. *Microb. Cell. Fact*. **13**, 2 (2014). 10.1186/1475-2859-13-2 PMC3897908 24397404

[2] Blombach B, Eikmanns BJ (2011). Current knowledge on isobutanol production with Escherichia coli, Bacillus subtilis and Corynebacterium glutamicum. Bioeng. Bugs.

[3] Li S, Wen J, Jia X (2011). Engineering Bacillus subtilis for isobutanol production by heterologous Ehrlich pathway construction and the biosynthetic 2-ketoisovalerate precursor pathway overexpression. Appl. Microbiol. Biotechnol..

[4] Connor MR, Liao JC (2008). Engineering of an Escherichia coli strain for the production of 3-methyl-1-butanol. Appl. Environ. Microbiol..

[5] Niu W, Molefe MN, Frost JW (2003). Microbial synthesis of the energetic material precursor 1,2,4-butanetriol. J. Am. Chem. Soc..

[6] Guterl JK (2012). Cell-free metabolic engineering: production of chemicals by minimized reaction cascades. ChemSusChem.

[7] Egan SE (1992). Molecular characterization of the Entner-Doudoroff pathway in Escherichia coli: sequence analysis and localization of promoters for the edd-eda operon. J. Bacteriol..

[8] Carsten JM, Schmidt A, Sieber V (2015). Characterization of recombinantly expressed dihydroxy-acid dehydratase from Sulfobus solfataricus-A key enzyme for the conversion of carbohydrates into chemicals. J. Biotechnol..

[9] Velasco JA (1993). Cloning of the dihydroxyacid dehydratase-encoding gene (ILV3) from Saccharomyces cerevisiae. Gene.

[10] Bromke MA (2013). Amino Acid Biosynthesis Pathways in Diatoms. Metabolites.

[11] Zhang C (2015). Dihydroxyacid dehydratase is important for gametophyte development and disruption causes increased susceptibility to salinity stress in Arabidopsis. J. Exp. Bot..

[12] Kanamori M, Wixom RL (1963). Studies in valine biosynthesis. V. Characteristics of the purified dihydroxyacid dehydratase from spinach leaves. J. Biol. Chem..

[13] Myers JW (1961). Dihydroxy acid dehydrase: an enzyme involved in the biosynthesis of isoleucine and valine. J. Biol. Chem..

[14] Conway T (1992). The Entner-Doudoroff pathway: history, physiology and molecular biology. FEMS Microbiol. Rev..

[15] Gottschalk G, Bender R (1982). D-Gluconate dehydratase from Clostridium pasteurianum. Methods Enzymol..

[16] Meloche HP, Wood WA (1964). The Mechanism of 6-Phosphogluconic Dehydrase. J. Biol. Chem..

[17] Stephens C (2007). Genetic analysis of a novel pathway for D-xylose metabolism in Caulobacter crescentus. J. Bacteriol..

[18] Weimberg R (1961). Pentose oxidation by Pseudomonas fragi. J. Biol. Chem..

[19] Andberg M (2016). Characterization and mutagenesis of two novel iron-sulphur cluster pentonate dehydratases. Appl. Microbiol. Biotechnol..

[20] Watanabe S, Shimada N, Tajima K, Kodaki T, Makino K (2006). Identification and characterization of L-arabonate dehydratase, L-2-keto-3-deoxyarabonate dehydratase, and L-arabinolactonase involved in an alternative pathway of L-arabinose metabolism. Novel evolutionary insight into sugar metabolism. J. Biol. Chem..

[21] Jiang Y (2015). Characterization of D-xylonate dehydratase YjhG from Escherichia coli. Bioengineered.

[22] Kim S, Lee SB (2008). Identification and characterization of the bacterial d-gluconate dehydratase in Achromobacter xylosoxidans. Biotechnol Bioproc E.

[23] Flint DH, Emptage MH, Finnegan MG, Fu W, Johnson MK (1993). The role and properties of the iron-sulfur cluster in Escherichia coli dihydroxy-acid dehydratase. J. Biol. Chem..

[24] Flint DH, Emptage MH (1988). Dihydroxy acid dehydratase from spinach contains a [2Fe-2S] cluster. J. Biol. Chem..

[25] Flint, D. H., Rothman, S. C., Suh, W. & Tomb, J. F. Identification and use of bacterial [2Fe-2S] dihydroxy-acid dehydratases, Patent WO2010037112 (A1) (2010).

[26] Rodriguez M, Wedd AG, Scopes RK (1996). 6-phosphogluconate dehydratase from Zymomonas mobilis: an iron-sulfur-manganese enzyme. Biochem. Mol. Biol. Int..

[27] Rahman MM (2017). The crystal structure of a bacterial L-arabinonate dehydratase contains a [2Fe-2S] cluster. ACS Chem. Biol..

[28] Krissinel E, Henrick K (2007). Inference of macromolecular assemblies from crystalline state. J. Mol. Biol..

[29] Scopes RK, Griffiths-Smith K (1984). Use of differential dye-ligand chromatography with affinity elution for enzyme purification: 6-phosphogluconate dehydratase from Zymomonas mobilis. Anal. Biochem..

[30] Hulo N (2006). The PROSITE database. Nucleic Acids Res..

[31] Rahman MM, Andberg M, Koivula A, Rouvinen J, Hakulinen N (2016). Crystallization and X-ray diffraction analysis of an L-arabinonate dehydratase from Rhizobium leguminosarum bv. trifolii and a D-xylonate dehydratase from Caulobacter crescentus. Acta Crystallogr. F. Struct. Biol. Commun..

[32] Kabsch W (2010). Integration, scaling, space-group assignment and post-refinement. Acta Crystallogr. D Biol. Crystallogr..

[33] McCoy AJ (2007). Phaser crystallographic software. J. Appl. Crystallogr..

[34] Emsley P, Lohkamp B, Scott WG, Cowtan K (2010). Features and development of Coot. Acta Crystallogr. D Biol. Crystallogr..

[35] Afonine PV (2012). Towards automated crystallographic structure refinement with phenix.refine. Acta Crystallogr. D Biol. Crystallogr..

[36] Chen VB (2010). MolProbity: all-atom structure validation for macromolecular crystallography. Acta Crystallogr. D Biol. Crystallogr..

[37] Buchanan CL, Connaris H, Danson MJ, Reeve CD, Hough DW (1999). An extremely thermostable aldolase from Sulfolobus solfataricus with specificity for non-phosphorylated substrates. Biochem. J..

[38] Clark K, Karsch-Mizrachi I, Lipman DJ, Ostell J, Sayers EW (2016). GenBank. Nucleic Acids Res..

[39] Sievers F (2011). Fast, scalable generation of high-quality protein multiple sequence alignments using Clustal Omega. Mol. Syst. Biol..

[40] Olson SA (1994). MacVector: an integrated sequence analysis program for the Macintosh. Methods Mol. Biol..

[41] Heinig M, Frishman D (2004). STRIDE: a web server for secondary structure assignment from known atomic coordinates of proteins. Nucleic Acids Res..

[42] Bond, C. S. & Schuttelkopf, A. W. ALINE: aWYSIWYG protein-sequence alignment editor for publication-quality alignments. *Acta Crystallogr. D Biol. Crystallogr.* **65**, 510–512 (2009). 10.1107/S0907444909007835 19390156

[43] The PyMOL Molecular Graphics System, Version 1. 8 Schrödinger, LLC, Portland, OR (2015).

