# Supplementary material for: The crystal structure of D-xylonate dehydratase reveals functional features of enzymes from the Ilv/ED dehydratase family
Source: Sci Rep. 2018 Jan 16;8:865. doi: 10.1038/s41598-018-19192-6 (PMC5770437; doi:10.1038/s41598-018-19192-6)
Supplement: Supplementary file 1 — Supplementary Information [file 41598_2018_19192_MOESM1_ESM.docx]

**SUPPLEMENTARY DATA**

**The crystal structure of D-xylonate dehydratase reveals functional features of enzymes from the Ilv/ED dehydratase family**

Mohammad Mubinur Rahman^1^, Martina Andberg^2^, Anu Koivula^2^, Juha Rouvinen^1^ and Nina Hakulinen^1*^

^1^ Department of Chemistry, University of Eastern Finland, PO Box 111, FIN-80101 Joensuu, Finland

^2^ VTT Technical Research Centre of Finland Ltd, PO Box 1000, FIN-02044 VTT, Espoo, Finland


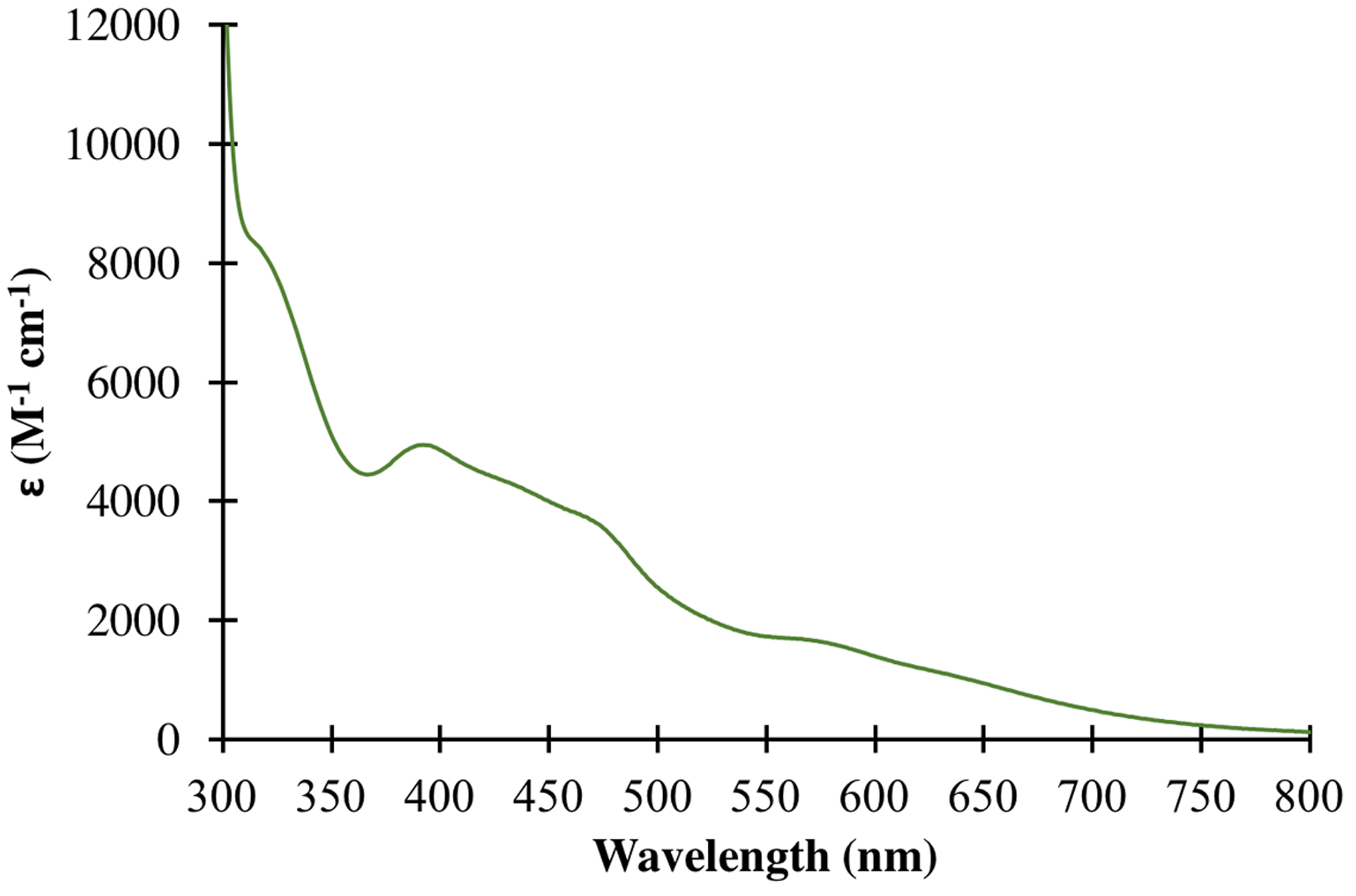


**Figure S1.** UV-Vis spectrum of *Cc*XyDHT. The spectrum shows the peaks that corresponding to

S →Fe (III) charge transfer bands at 320-330 nm, 387-397 nm, 470-480 nm, and at 570-580 nm.
